# Supplementary material for: Retrovirus insertions in host transcripts trigger de novo piRNA immunity
Source: EMBO J. 2026 May 2;45(11):3833–58. doi: 10.1038/s44318-026-00777-1 (PMC13226689; doi:10.1038/s44318-026-00777-1)
Supplement: Supplementary file 1 — Appendix [file 44318_2026_777_MOESM1_ESM.pdf]

APPENDIX

Retrovirus insertions in host transcripts trigger de novo piRNA immunity

Baptiste Rafanel[1,2], Liudmila Protsenko[1,2], Dominik Handler[1], Julius Brennecke[1,*], Kirsten-André Senti[1,*]

[1] Institute of Molecular Biotechnology of the Austrian Academy of Sciences (IMBA), Vienna BioCenter (VBC); Dr. Bohr-Gasse 3, 1030 Vienna, Austria.

[2] Vienna BioCenter PhD Program, Doctoral School of the University of Vienna and Medical University of Vienna, Vienna, Austria

[*] Correspondence: julius.brennecke@imba.oeaw.ac.at, senti@imba.oeaw.ac.at

Appendix Figure S1 **2**

Appendix Figure S2 **3**

Appendix Figure S3 **5**

Appendix Figure S4 **8**

Appendix Figure S5 **9**

Appendix Figure S6 **10**

Appendix Figure S7 **11**

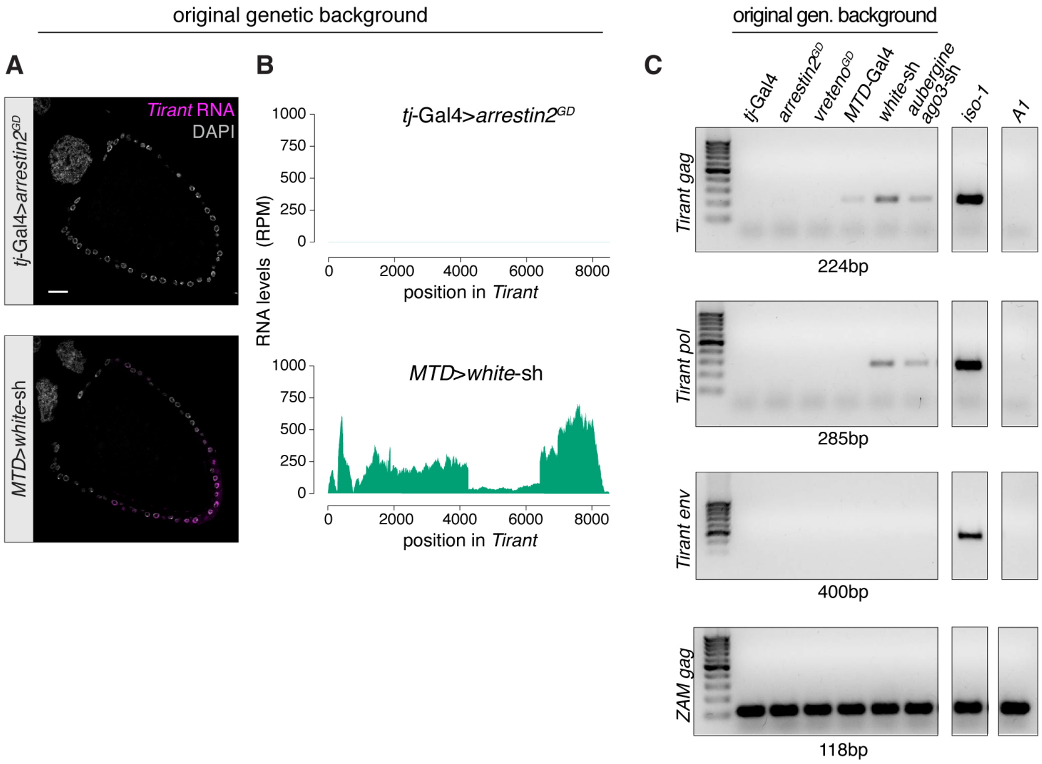

Appendix Figure S1: *Tirant* is absent or defective in laboratory strains used in previous piRNA studies.

(A) RNA-FISH detecting *Tirant* sense transcripts (magenta) in ovaries of the original soma (top) and germline (bottom) RNAi knockdown experiments (control conditions are shown). DNA staining (DAPI) in grey. Scale bar: 20µm.

(B) Density of polyA-selected Illumina RNA-seq reads (reads per million; RPM) mapping to the *Tirant* consensus sequence from the experimental conditions described in (A) (data from Senti *et al*, 2025).

(C) DNA gel electrophoresis images showing the result of PCR genotyping of genomic DNA of the laboratory strains used in this study to detect presence of *Tirant gag*, *pol*, or *env*. DNA from *iso-1* was used as a *Tirant*-containing positive control, and the *Tirant*-free DSPR founder strain *A1* was used as negative control. The *ZAM* PCR amplicon served as PCR positive control for all strains. DNA marker: 100bp DNA ladder.

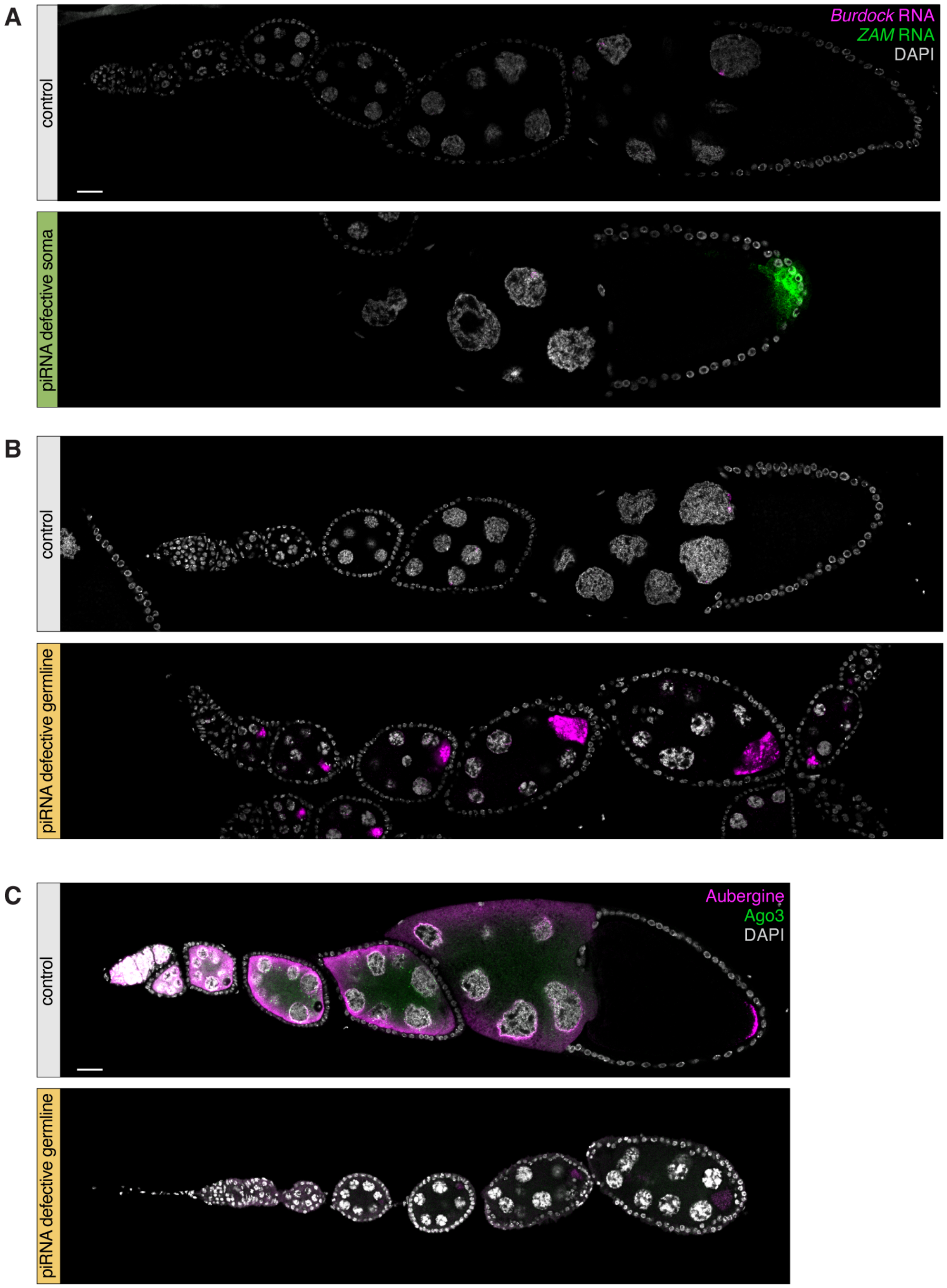

Appendix Figure S2: Verification of piRNA pathway knockdown efficiency using *iso-1* introgressed genotypes (used in Fig 3)

(A) RNA-FISH detection of *Burdock* (magenta) and *ZAM* sense transcripts (green) in ovarioles from control ovaries (*arrestin2* knockdown; top) and somatic piRNA pathway–deficient ovaries (*vreteno* knockdown; bottom). Somatic piRNA pathway–deficient ovarioles show *ZAM* expression only, indicating transposable element derepression in somatic cells.

(B) RNA-FISH of *Burdock* (magenta) and *ZAM* sense transcripts (green) in ovarioles with germline-specific RNAi knockdown of a control gene (*white*; top) or germline piRNA pathway components

(*aubergine* and *ago3*; bottom). Germline piRNA pathway–deficient ovarioles show *Burdock*-only expression.

(C) Immunofluorescence against Aubergine (magenta) and Ago3 (green) in ovarioles with germline specific RNAi knockdown of a control gene (*white;* top) or germline piRNA pathway factors (*aubergine* and *ago3*; bottom). Aubergine and Ago3 signals are lost upon combined knockdown, confirming knockdown efficiency.

In all images DNA is stained with DAPI (grey). Scale bar: 20μm.

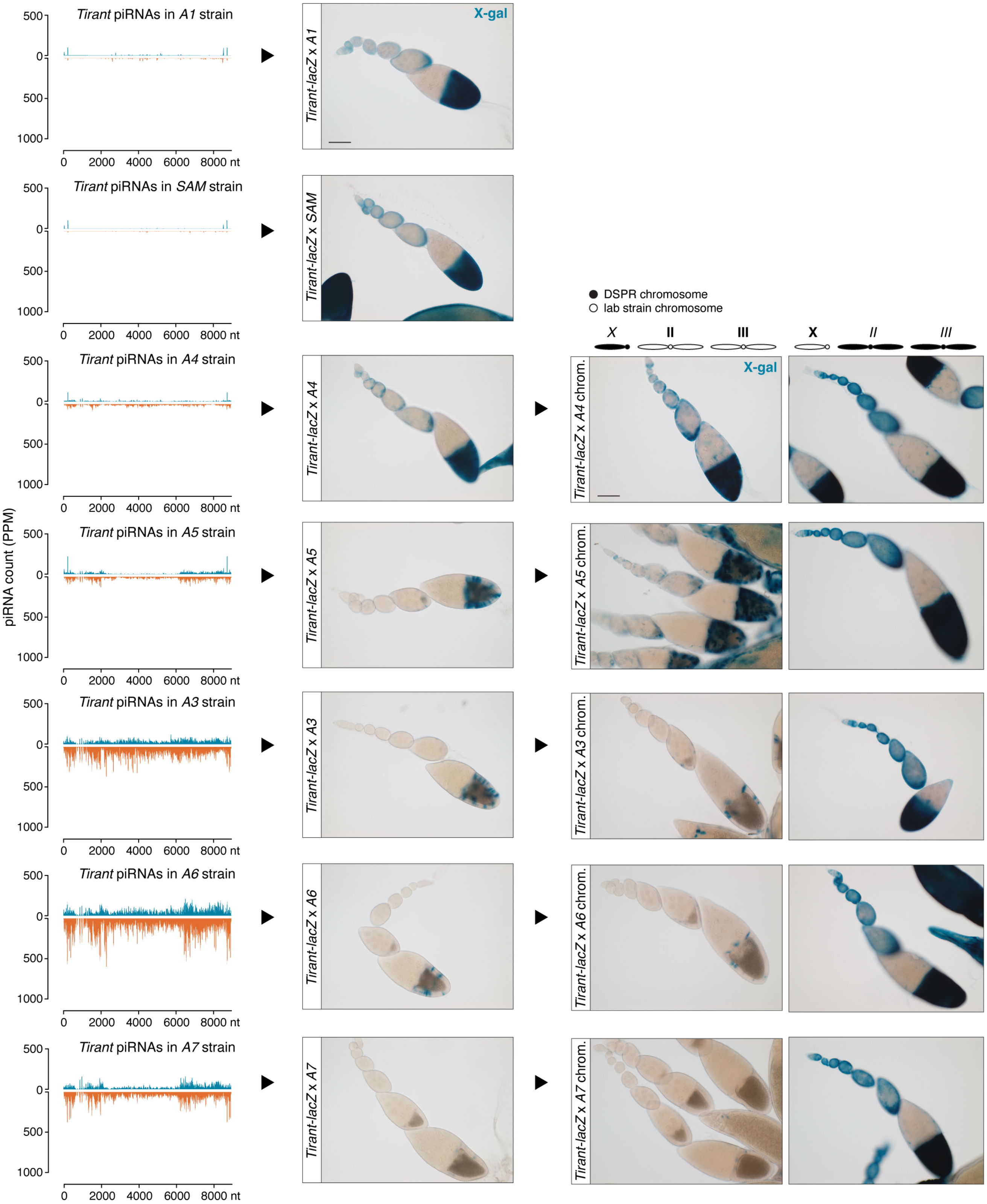

Tirant piRNAs in A1 strain
Tirant piRNAs in SAM strain
Tirant piRNAs in A4 strain
Tirant piRNAs in A5 strain
Tirant piRNAs in A3 strain
Tirant piRNAs in A6 strain
Tirant piRNAs in A7 strain
piRNA count (PPM)
500
0
500
1000
0 2000 4000 6000 8000 nt
X-gal
Tirant-lacZ x A1
Tirant-lacZ x SAM
Tirant-lacZ x A4
Tirant-lacZ x A5
Tirant-lacZ x A3
Tirant-lacZ x A6
Tirant-lacZ x A7
DSPR chromosome
lab strain chromosome
X II III X II III
Tirant-lacZ x A4 chrom.
Tirant-lacZ x A5 chrom.
Tirant-lacZ x A3 chrom.
Tirant-lacZ x A6 chrom.
Tirant-lacZ x A7 chrom.

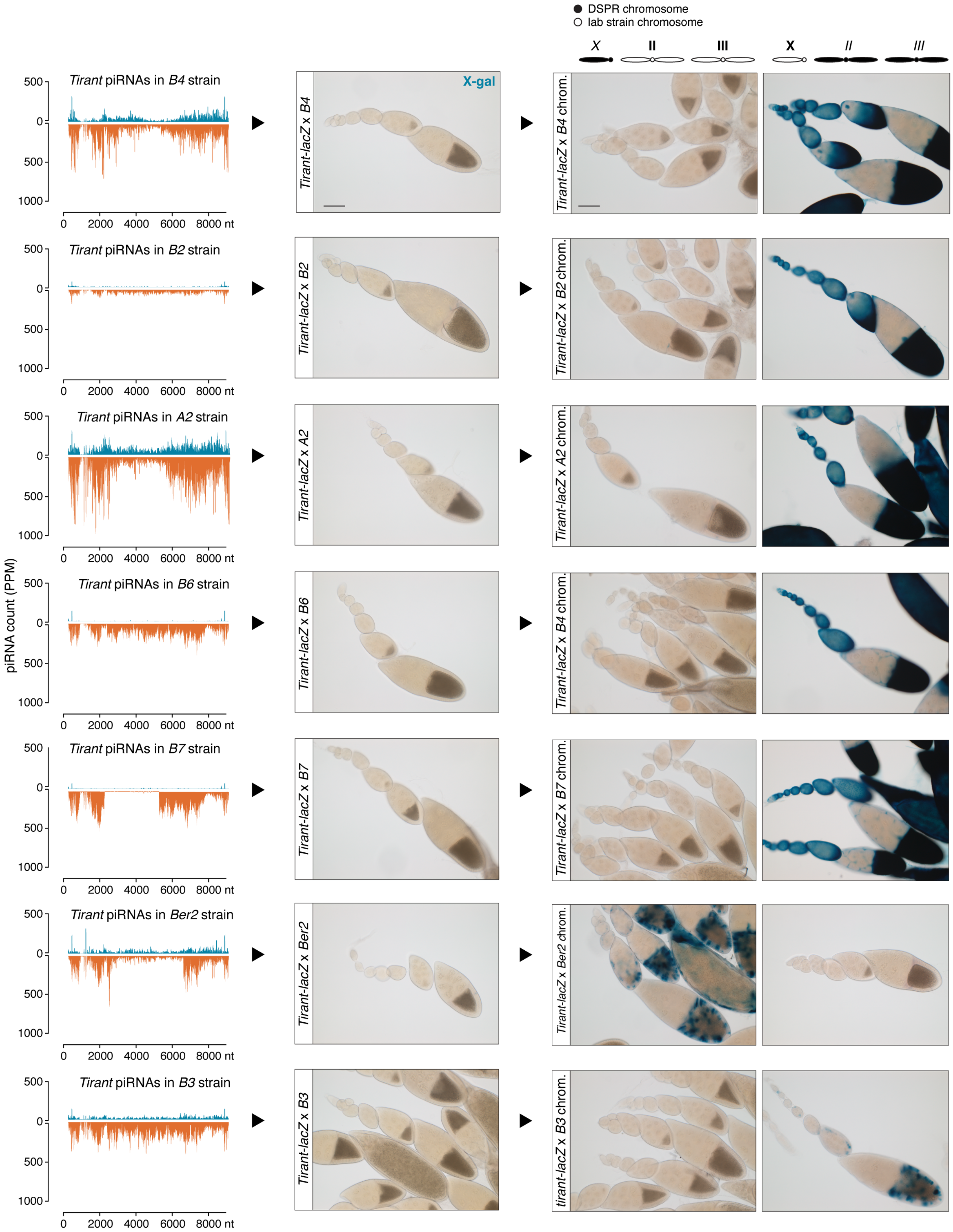
DSPR chromosome
lab strain chromosome
X
II
III
X
II
III
piRNA count (PPM)
Tirant piRNAs in B4 strain
Tirant piRNAs in B2 strain
Tirant piRNAs in A2 strain
Tirant piRNAs in B6 strain
Tirant piRNAs in B7 strain
Tirant piRNAs in Ber2 strain
Tirant piRNAs in B3 strain
500
0
500
1000
0 2000 4000 6000 8000 nt
X-gal
Tirant-lacZ x B4
Tirant-lacZ x B2
Tirant-lacZ x A2
Tirant-lacZ x B6
Tirant-lacZ x B7
Tirant-lacZ x Ber2
Tirant-lacZ x B3
Tirant-lacZ x B4 chrom.
Tirant-lacZ x B2 chrom.
Tirant-lacZ x A2 chrom.
Tirant-lacZ x B4 chrom.
Tirant-lacZ x B7 chrom.
Tirant-lacZ x Ber2 chrom.
tirant-lacZ x B3 chrom.

Appendix Figure S3: Systematic analysis of *Tirant* silencing capacity in DSPR strains and contributions of the X chromosome and autosomes

Shown are the X-gal stainings (*Tirant*-lacZ) of ovarioles summarized in Fig 3D and the respective profiles of piRNAs mapping to *Tirant* in each DSPR strain. piRNA density plots and X-gal stainings from *A1*, *B2*, and *A6* are the same as in Fig 3 and displayed for comparison. Scale bar: 100µm.

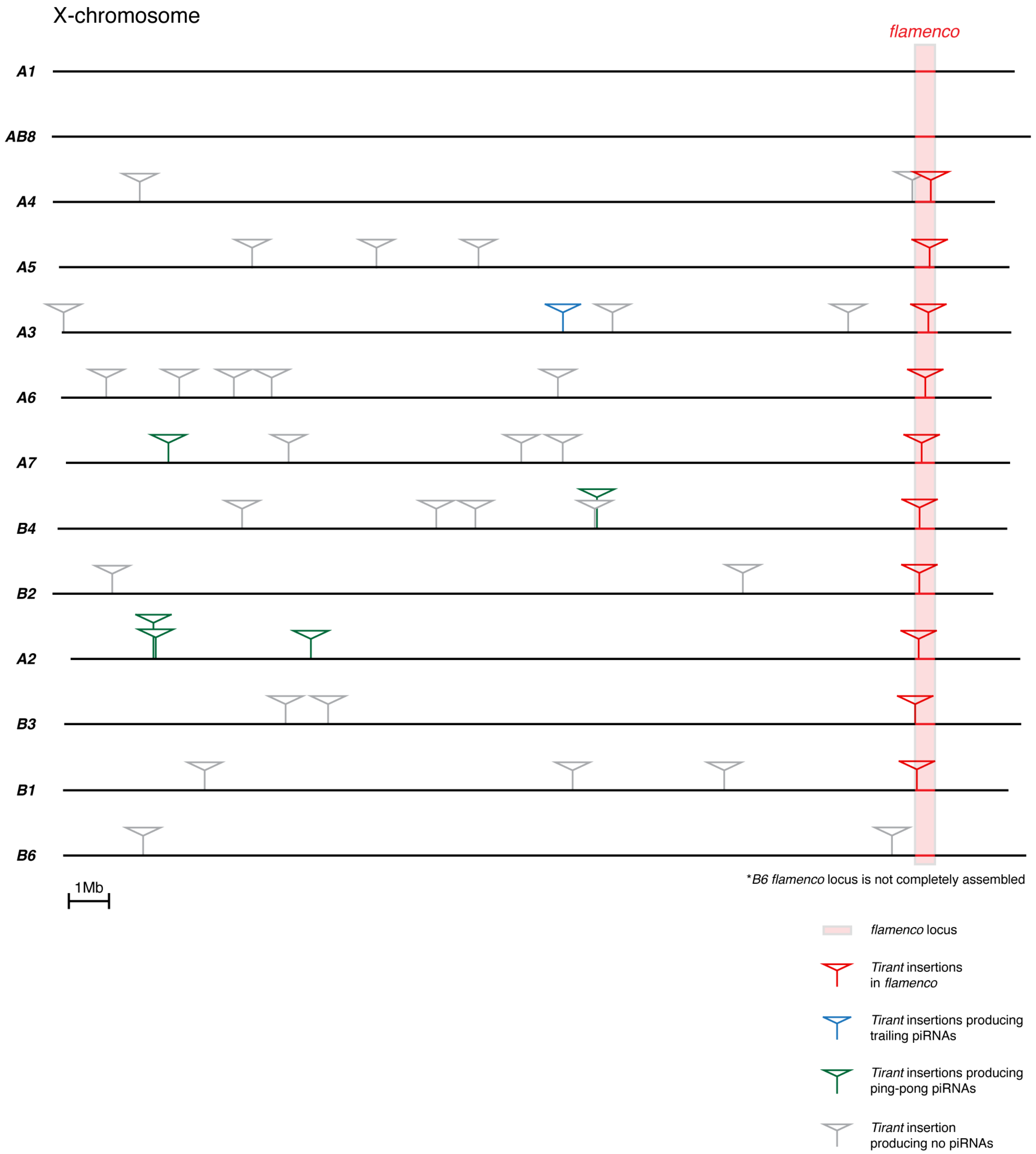

Appendix Figure S4: Overview of all X-chromosomal *Tirant* insertions in DSPR strains.
Schematic of X-chromosomal *Tirant* insertion sites in each DSPR strain based on long-read genome assemblies (Chakraborty *et al*, 2019). Genome-unique piRNA patterns flanking insertion sites were visually inspected to classify insertions as producing ping-pong piRNAs (green; strains *A7*, *B4*, and *A2*), uni-stranded trailing piRNAs (blue; *A3*), insertions within *flamenco* (red), or insertions lacking surrounding piRNAs (grey). Scale bar= 1 Mb.

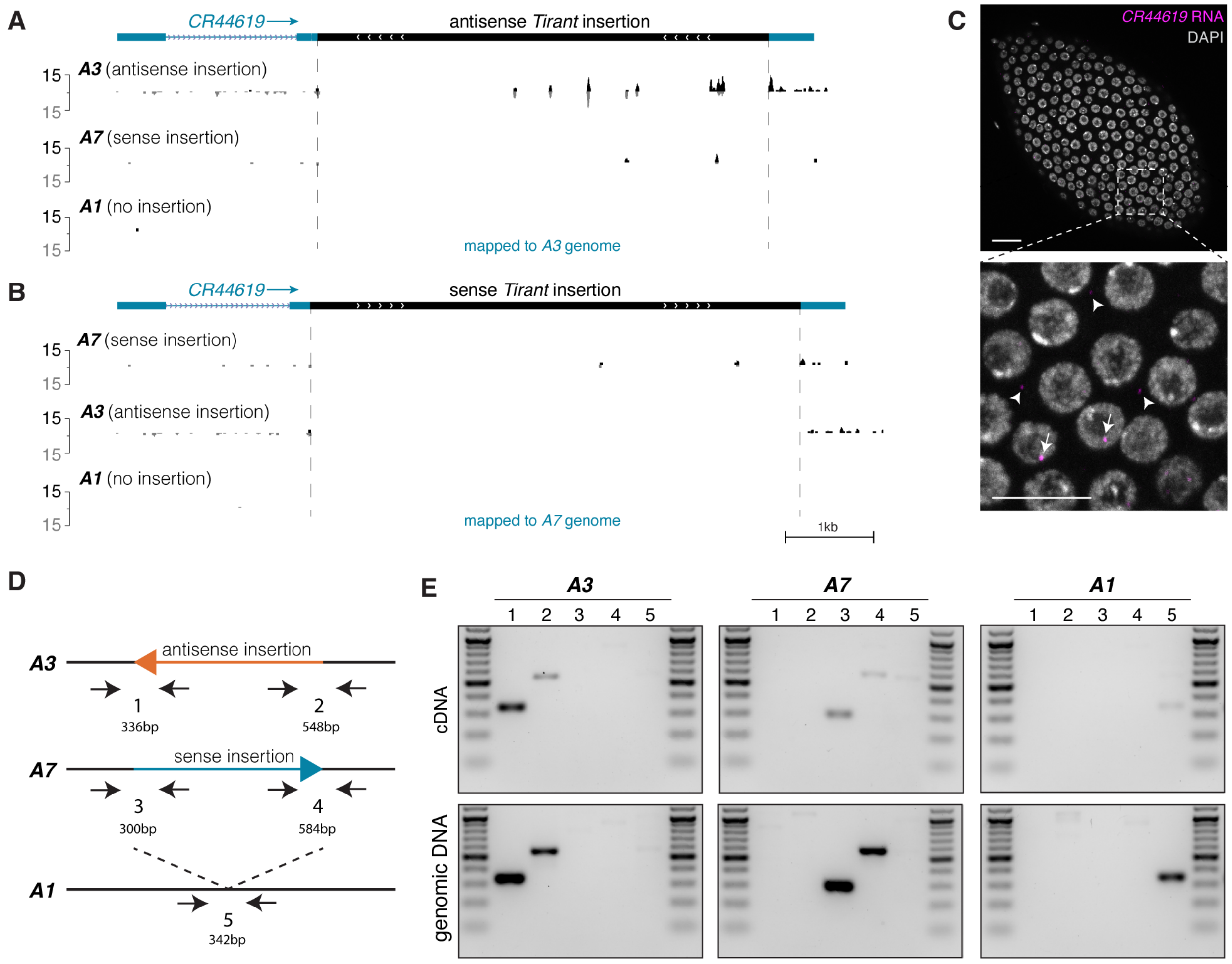

Appendix Figure S5: A *Tirant* insertion in the long non-coding RNA locus *CR44619* generates low amounts of piRNAs, preferentially in the antisense orientation.

(A) UCSC browser screenshot of the *CR44619* locus in the *A3* genome. The three tracks show genome-unique piRNAs (in PPM) from ovaries of the *A3* (top), *A7* (middle), or *A1* strain (bottom).

(B) As in panel A, but with genome-unique piRNAs mapped to the *A7* genome.

(C) Maximum intensity projection of RNA-FISH signal detecting *CR44619* transcripts (magenta) in a stage 8 egg chamber. The image below is a zoom-in of the boxed part in the top panel. Arrows indicate *CR44619* signal in the nucleus, and arrowheads signal in the cytoplasm. DNA (DAPI) is shown in grey. Scale bar: 20µm.

(D) Schematic showing the positions of PCR amplicons and their expected sizes used in panel E to test the presence of the *Tirant* sequence as part of the *CR44619* RNA in the *A3*, *A7*, and *A1* strains.

(E) RT-PCR on cDNA and PCR on genomic DNA from *A1* and *B3* to detect chimeric transcripts between *CR44619* and *Tirant*. Positions of PCR amplicons are shown in D. Marker: 100bp DNA ladder.

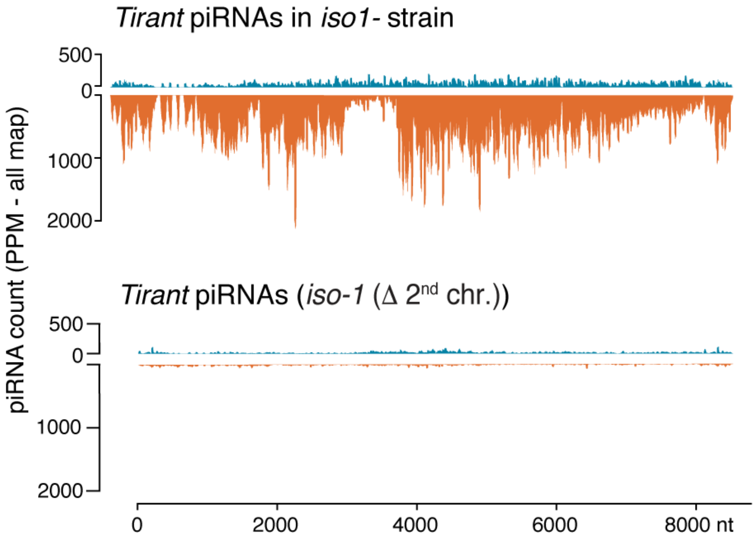

Appendix Figure S6: The *iso-1*$^{\Delta\ 2nd\ chr}$ strain does not produce piRNAs against *Tirant.*
Density plot showing piRNAs (in PPM) mapping to the *Tirant* consensus sequence from ovaries of the *iso-1* strain (top) and from the *iso-1(Δ2*nd *chr.*) strain (bottom). The top plot is reused from Fig 5B for comparison.

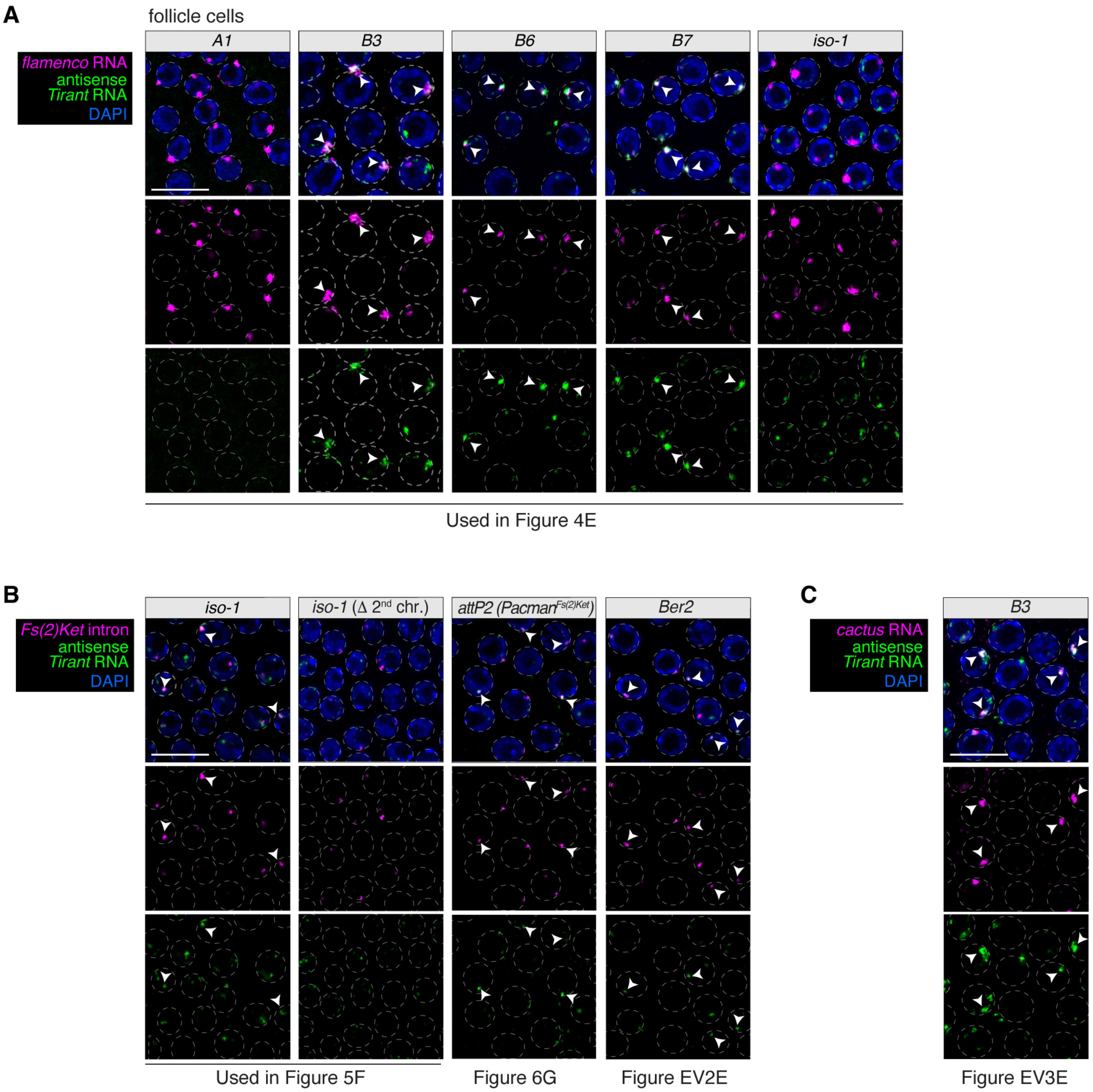

Appendix Figure S7: Single channel images of multiplex RNA-FISH experiments used in this study
(A) RNA-FISH detecting *flamenco* (magenta) and antisense *Tirant* transcripts (green) in follicle cells of stage 8 egg chambers from indicated genotypes, with the exception of the *B3* strain in which nuclei from a stage 10A egg chamber is shown. Circumferences of follicle cell nuclei, based on DAPI staining, are marked by dashed lines. Arrowheads indicate foci containing both FISH signals. Scale bar: 20µm.
(B) RNA-FISH detecting intronic *Fs(2)Ket* (magenta) and antisense *Tirant* (green) transcripts in follicle cells of stage 8 egg chambers from indicated genotypes. Scale bar: 20µm.
(C) RNA-FISH detecting *cactus* (magenta) and antisense *Tirant* (green) transcripts in follicle cells from stage 10A egg chambers from indicated genotypes. Scale bar: 20µm.
