# Supplementary material for: Retrovirus insertions in host transcripts trigger de novo piRNA immunity
Source: EMBO J. 2026 May 2;45(11):3833–58. doi: 10.1038/s44318-026-00777-1 (PMC13226689; doi:10.1038/s44318-026-00777-1)
Supplement: Supplementary file 3 — Dataset EV1 [file 44318_2026_777_MOESM3_ESM.zip › Dataset EV1 readme.rtf]

Dataset EV1: Detailed experimental resultsSheet 1 - Fig 1 EV1: piRNA PPM, ping-pong score, ping-pong Z score and phasing Z score used in Figure 1 and EV1 to generate the heatmaps and the statistical analyses. Sheet 2 - Fig 4 Tirant insertions in flam: Genomic coordinates of every Tirant insertions found in the DSPR genomes and whether they act as a source of piRNAsSheet 3 - Fig S4 X chr Tirant insertions : Tirant insertions in flamenco and their distances from the Transcriptional start site of flamencoSheet 4 - Fig 6 - RT-qPCR: RT-qPCR data used in Figure 6ESheet 5 - Figure EV4 - Fertility assay: Data used for the fertility assay in Figure EV4
