# Supplementary material for: Retrovirus insertions in host transcripts trigger de novo piRNA immunity
Source: EMBO J. 2026 May 2;45(11):3833–58. doi: 10.1038/s44318-026-00777-1 (PMC13226689; doi:10.1038/s44318-026-00777-1)
Supplement: Supplementary file 4 — Dataset EV2 [file 44318_2026_777_MOESM4_ESM.zip › Dataset EV2 readme.rtf]

Dataset EV2: Detailed experimental reagents and tools used in this studySheet 1: Drosophila melanogaster strains used Sheet 2: Primer sequences for generating DNA constructs and RT-PCR/RT-qPCRSheet 3: DNA constructs generated for this study with DNA templates and primersSheet 4: Sequences of HCR FISH Probes used Sheet 5: Antibodies used with IF dilutionsSheet 6: Sequences used to generate the aub/ago3 double sh lineSheet 7: Public sequencing datasets used in this study
