# Supplementary figures and images for: Retrovirus insertions in host transcripts trigger de novo piRNA immunity
Source: EMBO J. 2026 May 2;45(11):3833–58. doi: 10.1038/s44318-026-00777-1 (PMC13226689; doi:10.1038/s44318-026-00777-1)

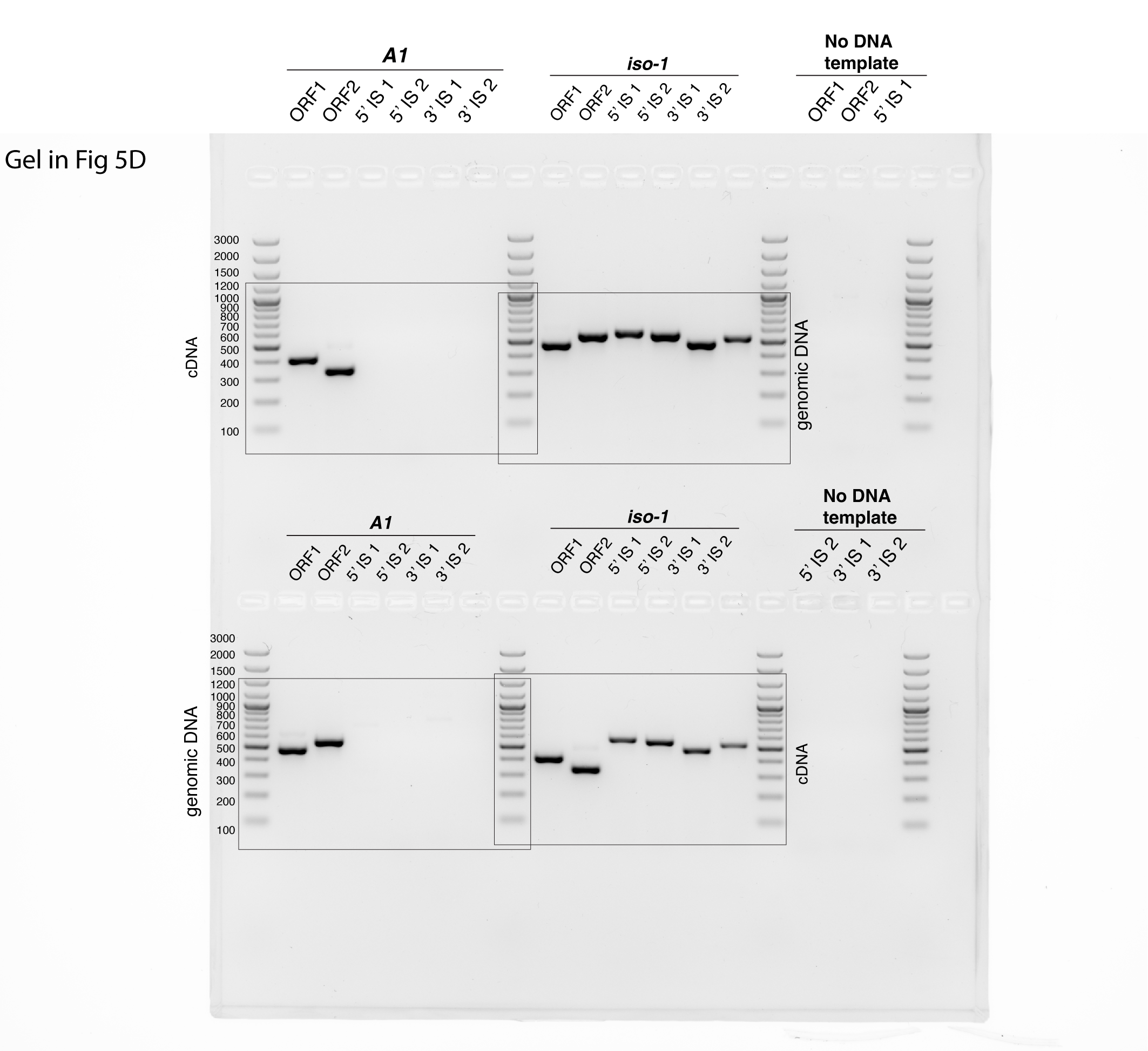

Supplement: Supplementary file 5 — Source data Fig. 5 [file 44318_2026_777_MOESM5_ESM.zip › Fig5D-RT-PCR-Ket-A1-Iso1-Annotated.tif]

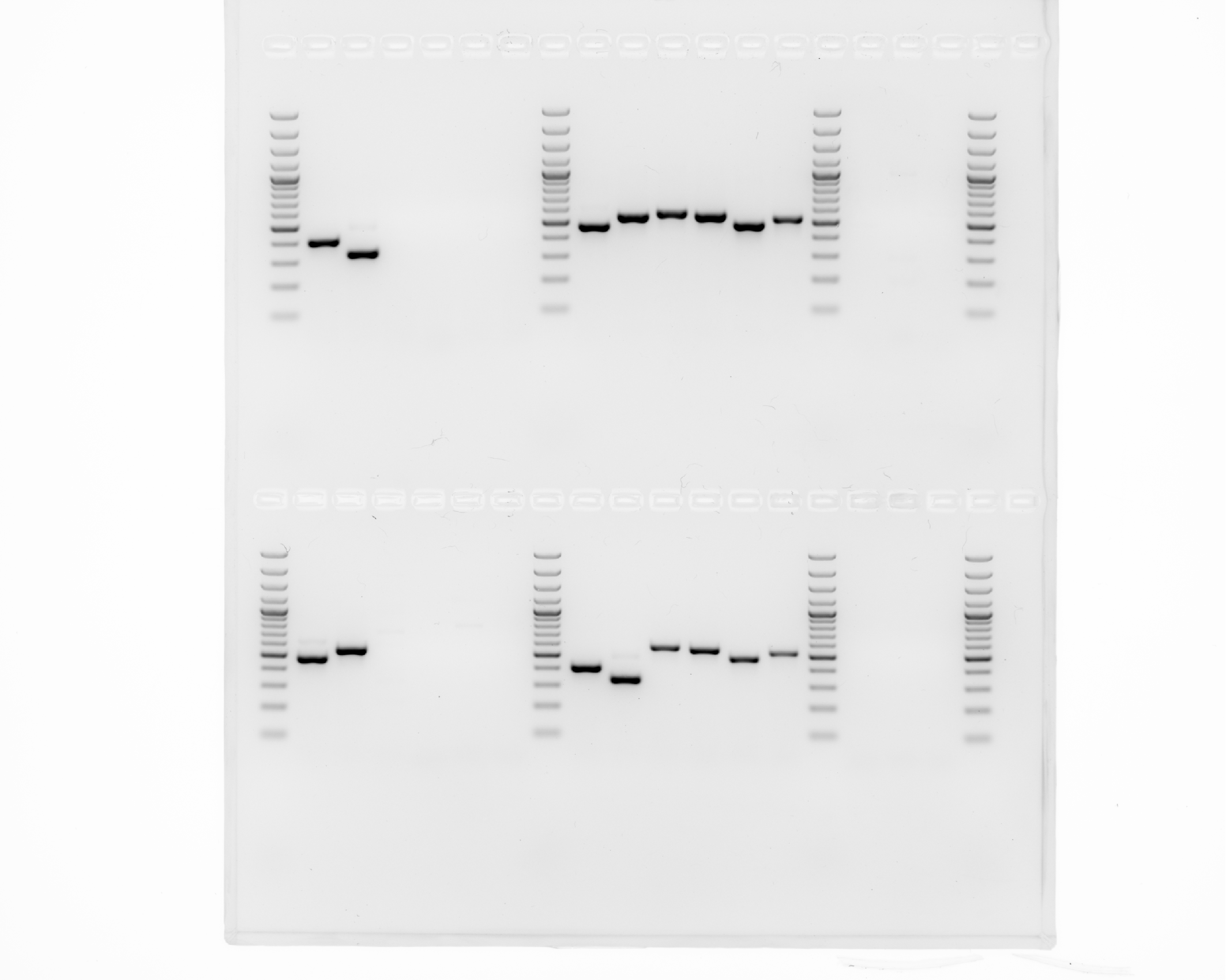

Supplement: Supplementary file 5 — Source data Fig. 5 [file 44318_2026_777_MOESM5_ESM.zip › Fig5D-RT-PCR-Ket-A1-Iso1-Unannotated.tif]

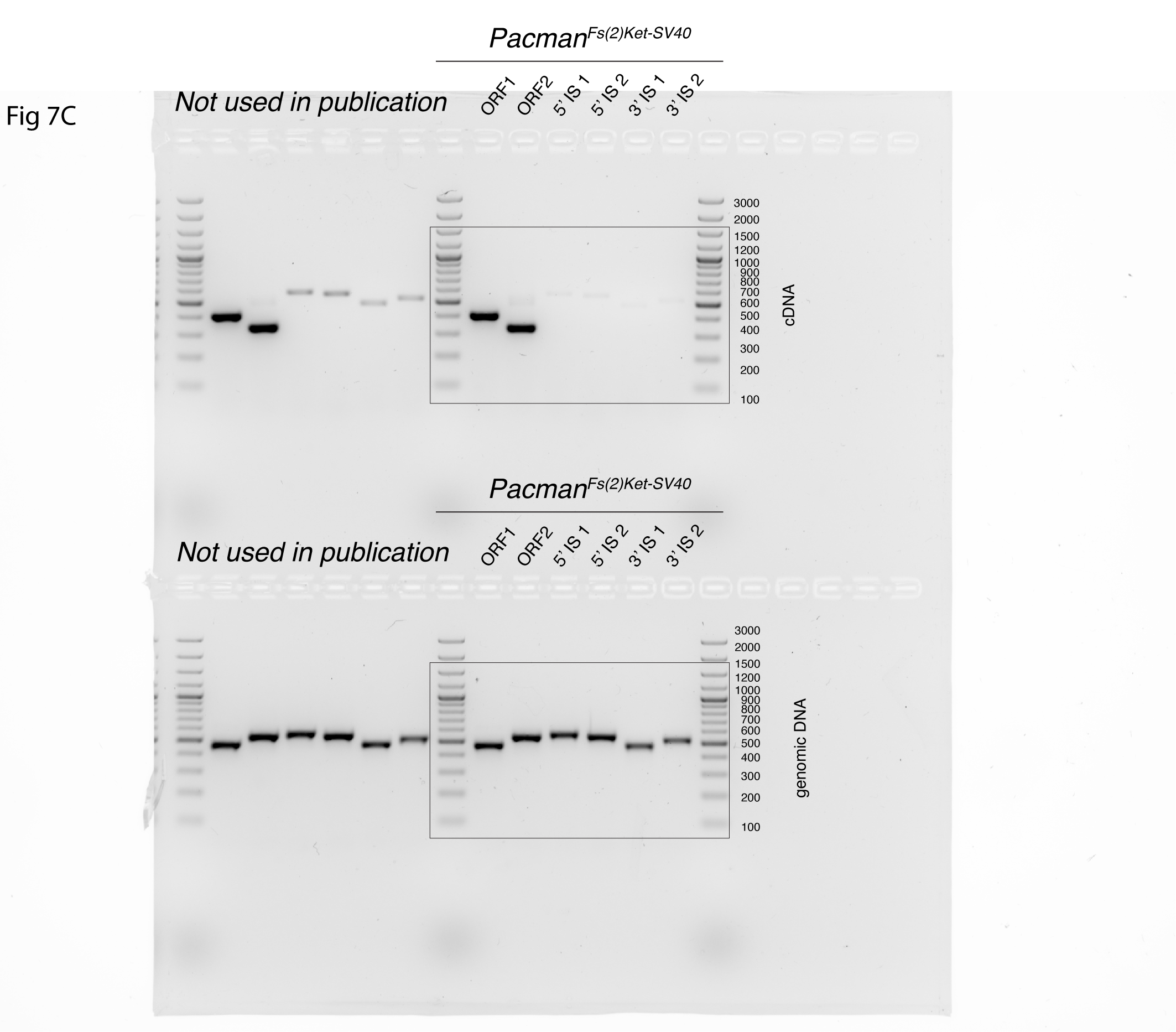

Supplement: Supplementary file 7 — Source data Fig. 7 [file 44318_2026_777_MOESM7_ESM.zip › Fig7C-RT-PCR-Ket-Ket-SV40-Annotated.tif]

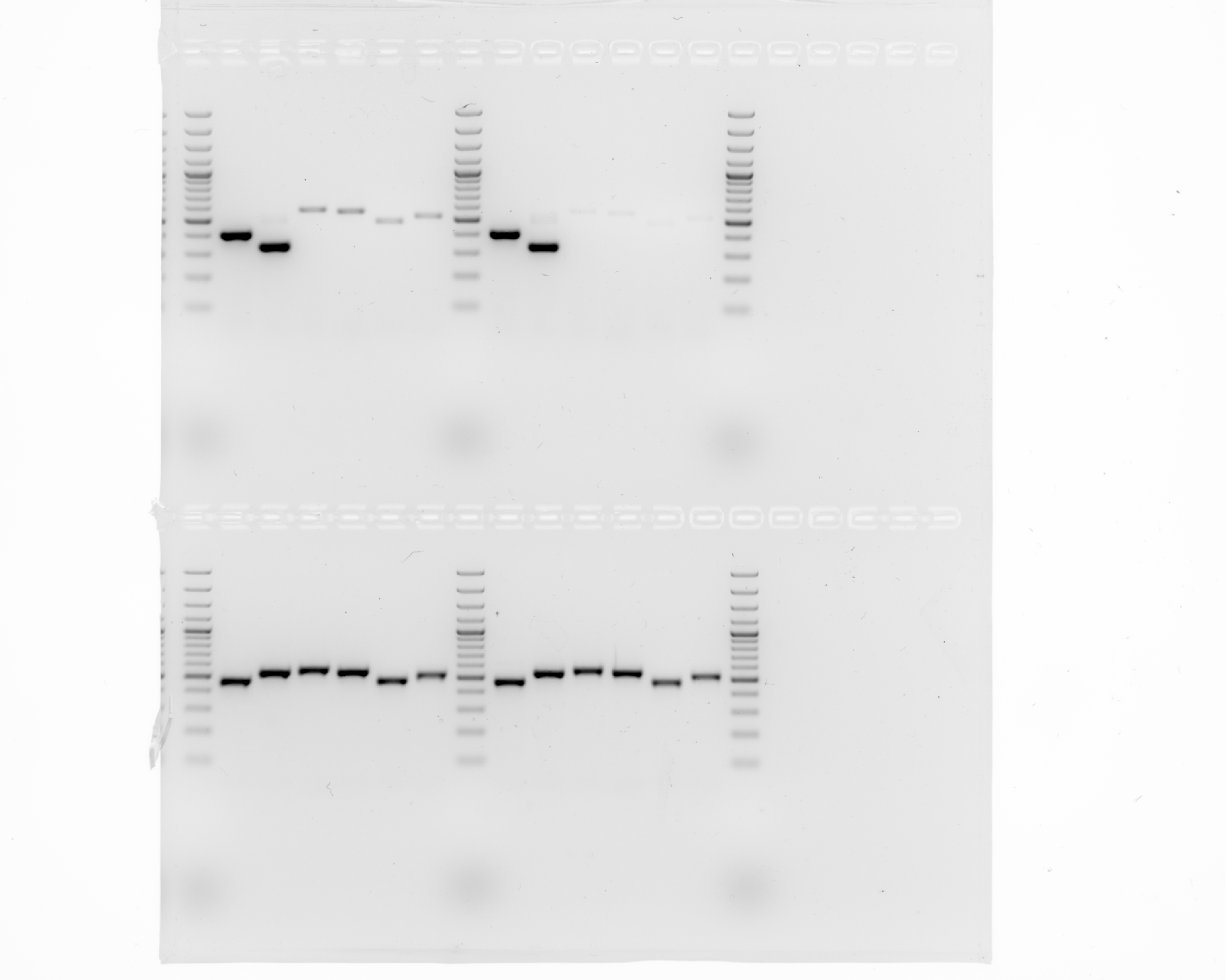

Supplement: Supplementary file 7 — Source data Fig. 7 [file 44318_2026_777_MOESM7_ESM.zip › Fig7C-RT-PCR-Ket-Ket-SV40-Unannotated.tif]

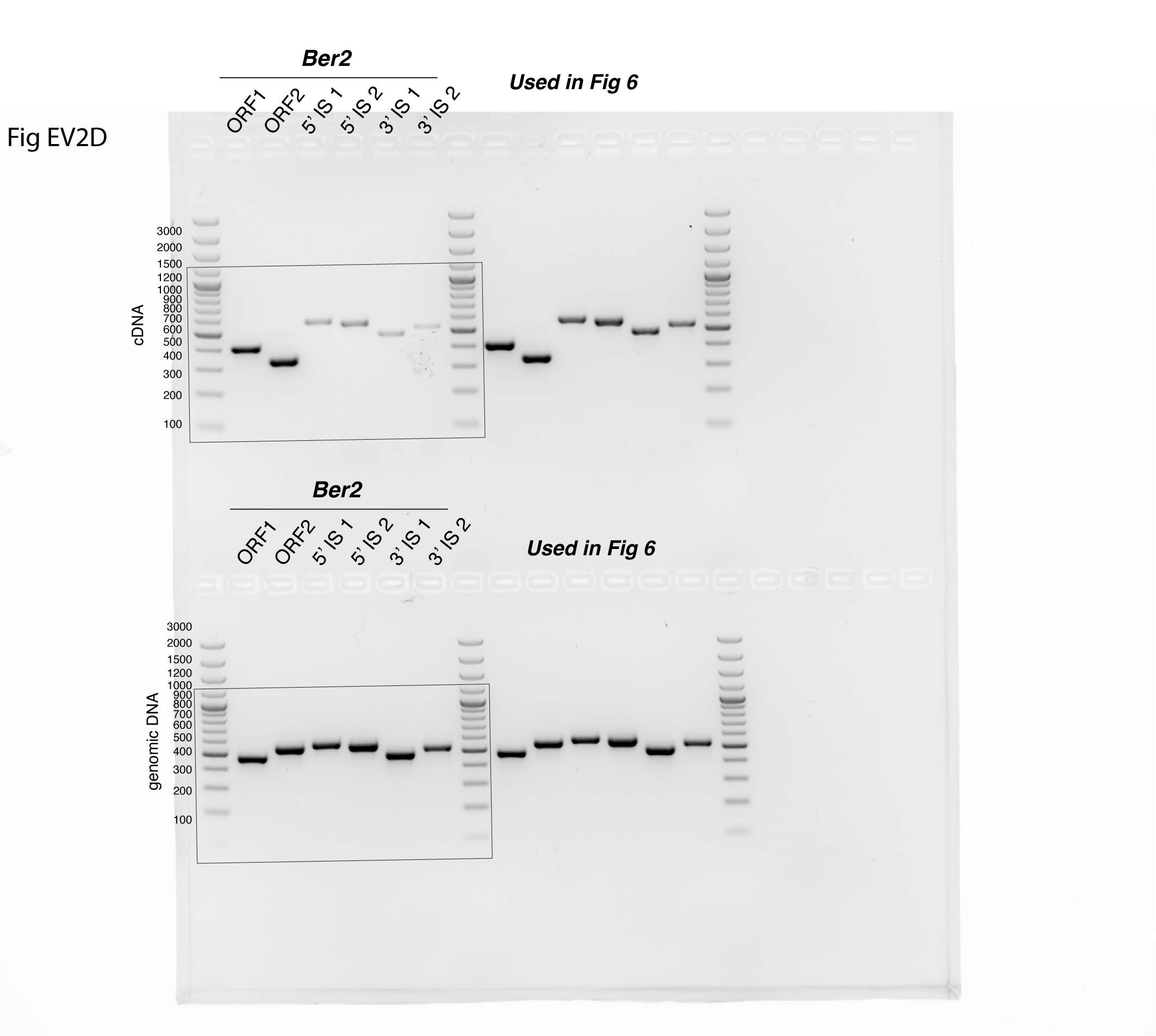

Supplement: Supplementary file 8 — Figure EV2 Source Data [file 44318_2026_777_MOESM8_ESM.zip › FigEV2D-RT-PCR-Ket-Ber2-Annotated.tif]

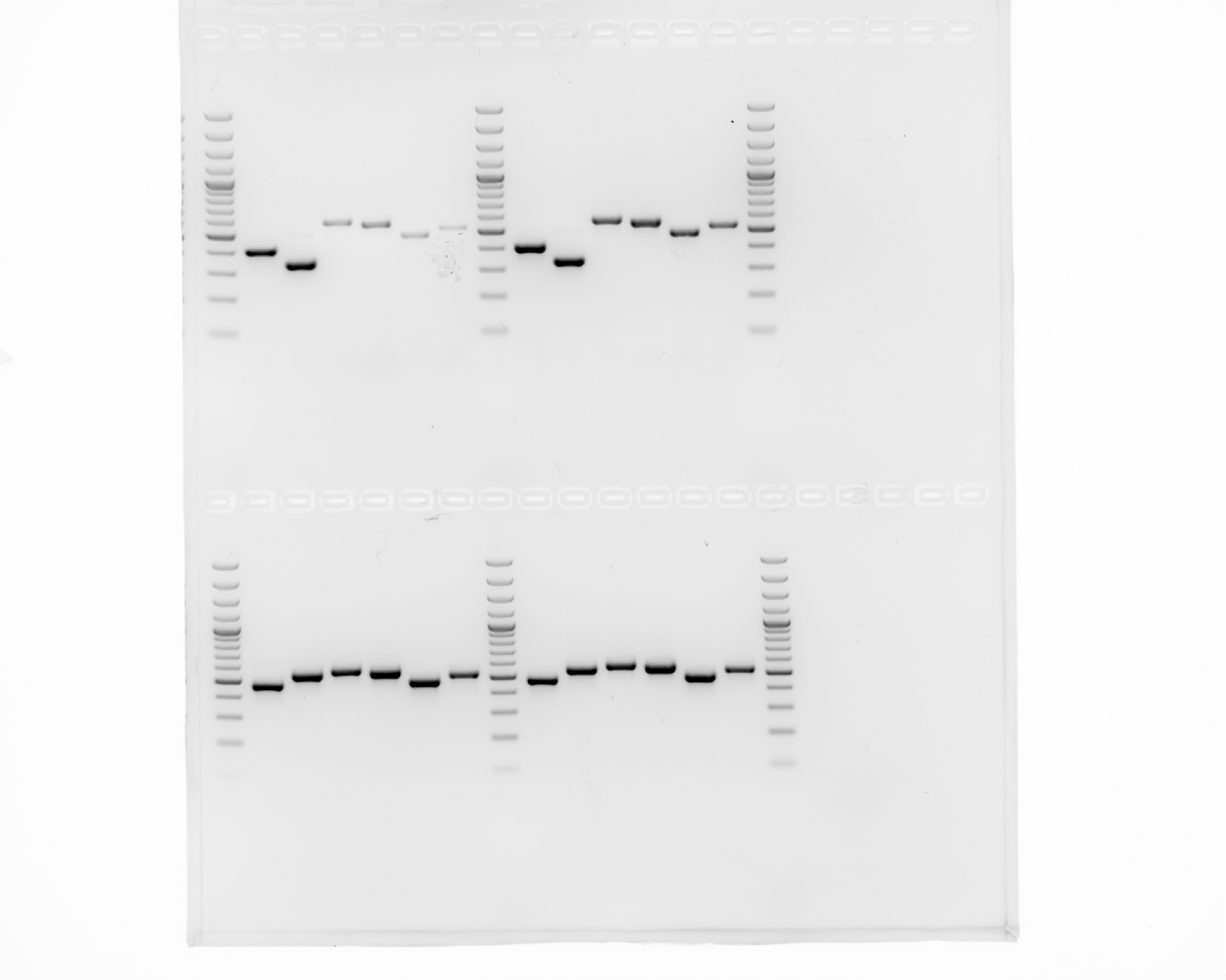

Supplement: Supplementary file 8 — Figure EV2 Source Data [file 44318_2026_777_MOESM8_ESM.zip › FigEV2D-RT-PCR-Ket-Ber2-Unannotated.tif]

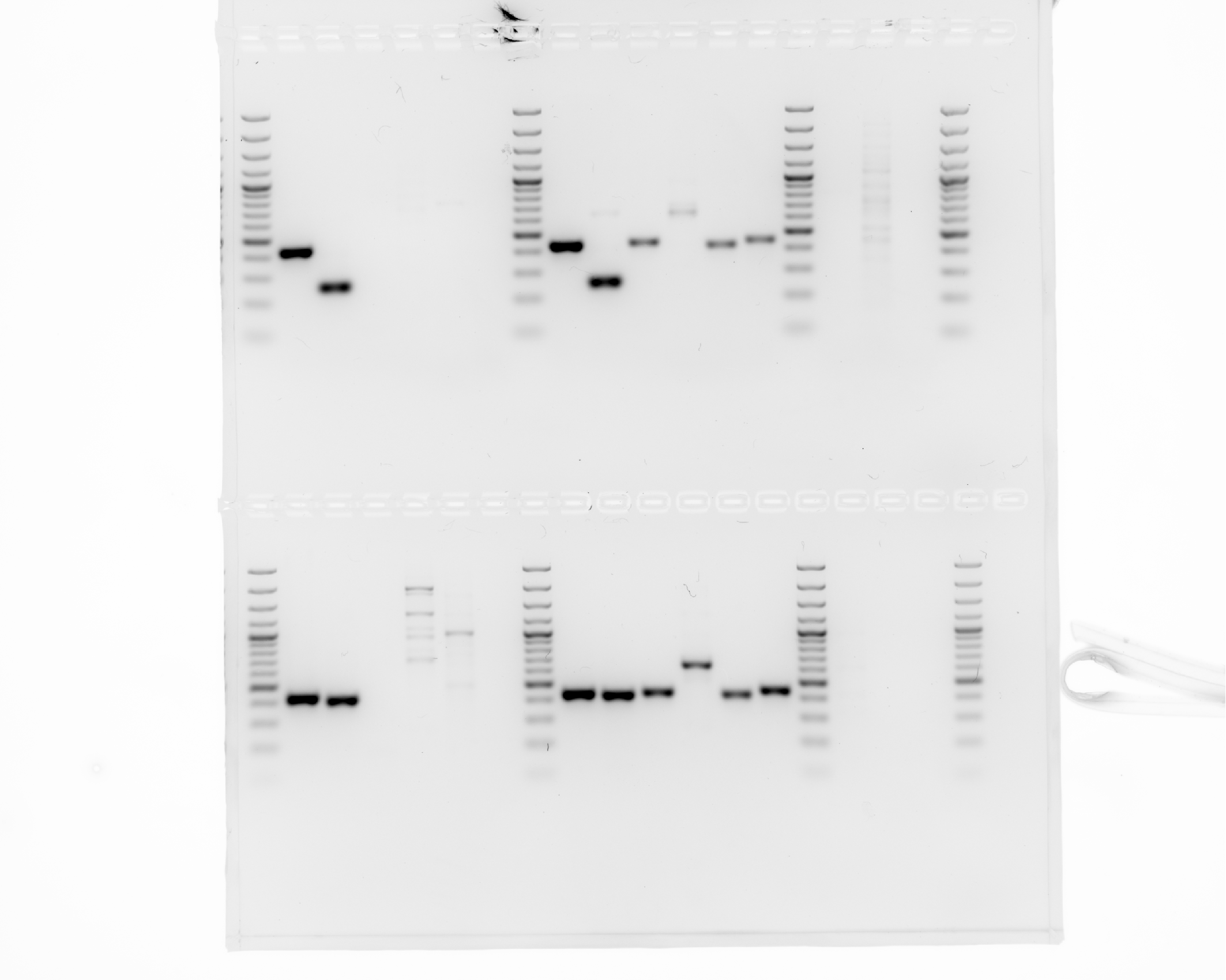

Supplement: Supplementary file 9 — Figure EV3 Source Data [file 44318_2026_777_MOESM9_ESM.zip › FigEV3D-RT-PCR-cact-A1-B3-Unannotated.tif]

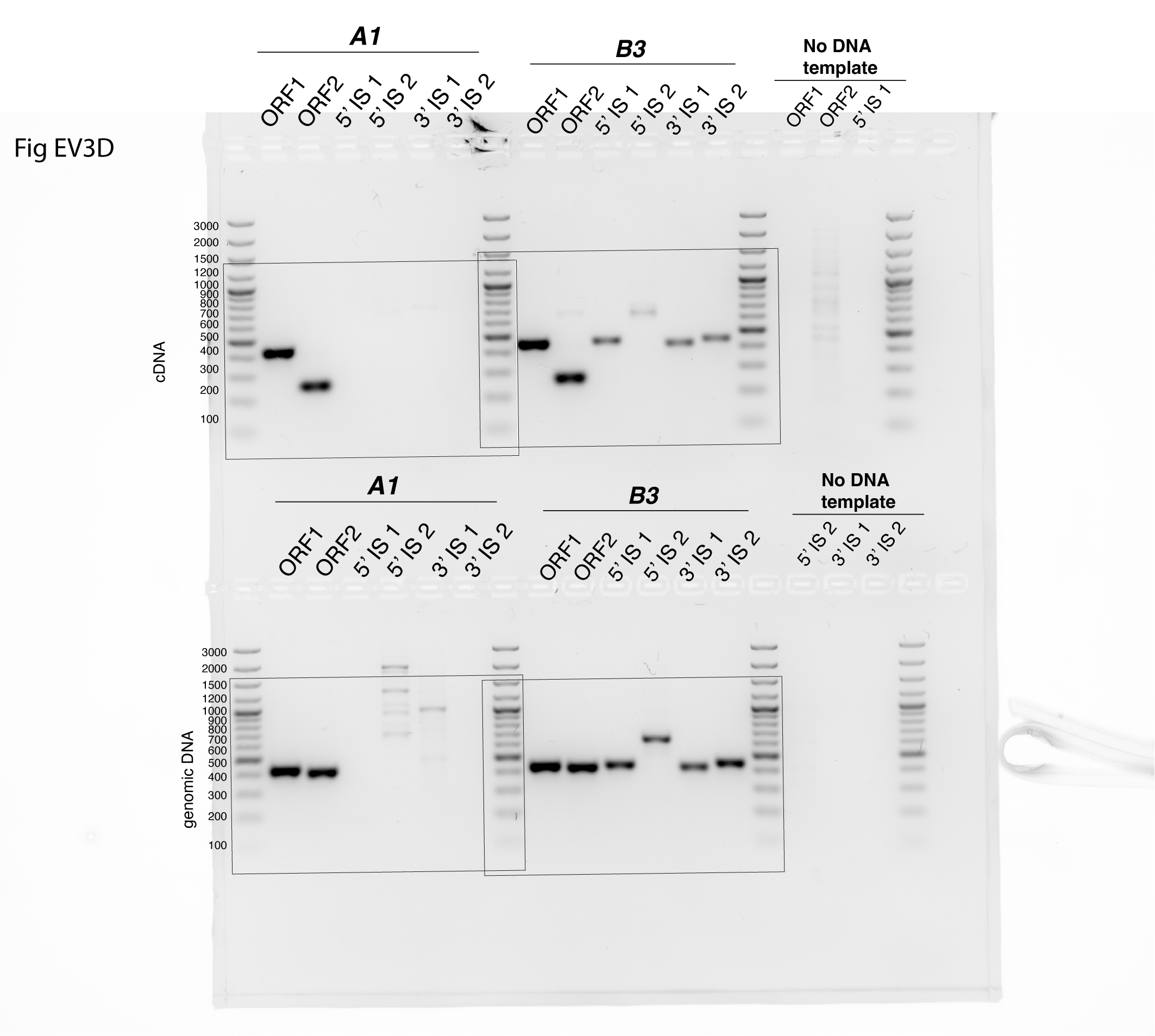

Supplement: Supplementary file 9 — Figure EV3 Source Data [file 44318_2026_777_MOESM9_ESM.zip › FigEV3D-RT-PCR-cact-A1-B3-Annotated.tif]
