# Supplementary material for: Retrovirus insertions in host transcripts trigger de novo piRNA immunity
Source: EMBO J. 2026 May 2;45(11):3833–58. doi: 10.1038/s44318-026-00777-1 (PMC13226689; doi:10.1038/s44318-026-00777-1)
Supplement: Supplementary file 11 — Expanded View Figures [file 44318_2026_777_MOESM11_ESM.pdf]

Expanded View Figures

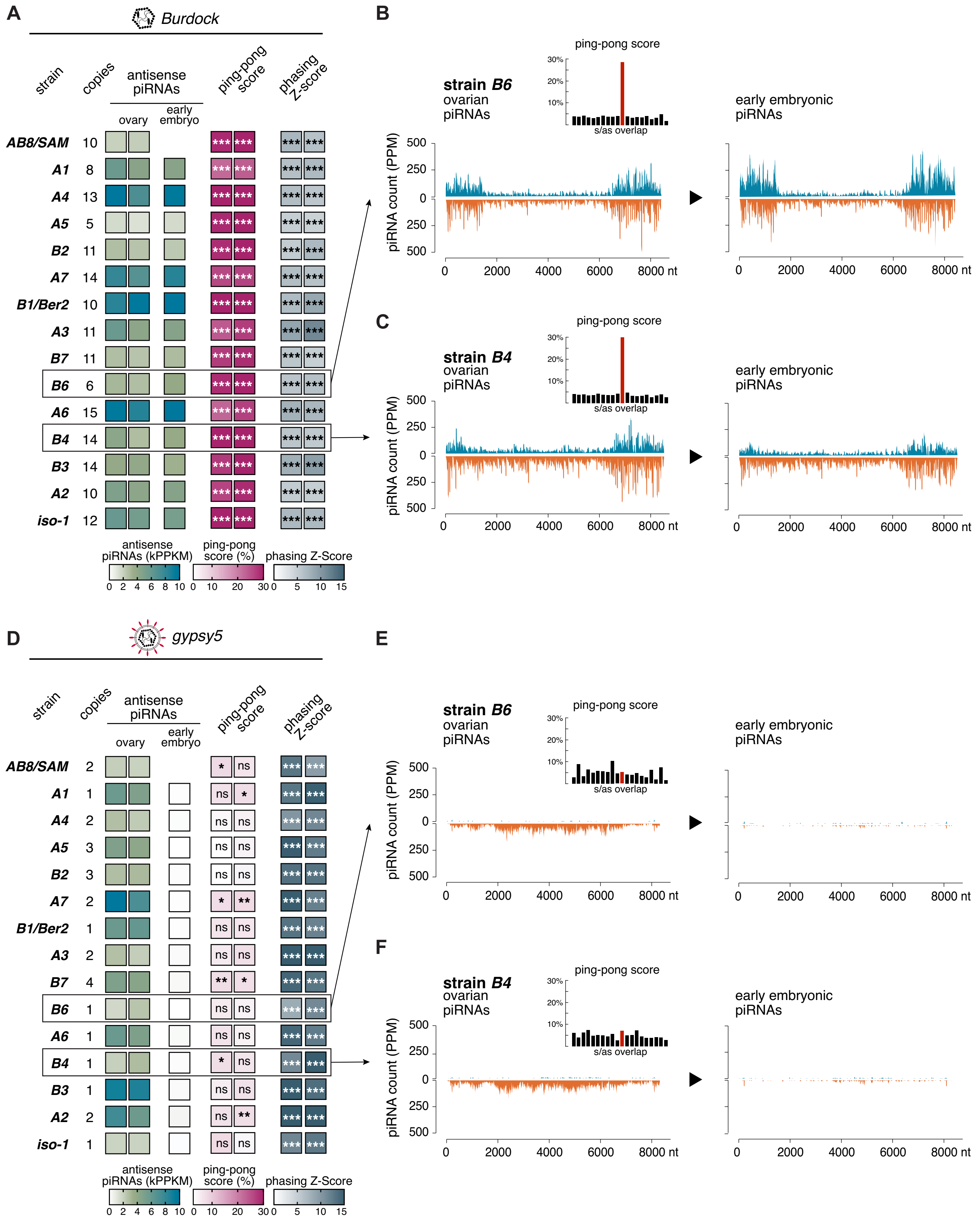

◀ **Figure EV1. Characteristics of piRNAs targeting *Burdock* (germline transposon) or *gypsy5* (somatic transposon) in DSPR founder strains.**

(**A**) Genomic copy numbers of *Burdock* (left) estimated from genomic Illumina data and heatmaps (right) of antisense piRNA levels equal or longer than 23 nucleotides (thousand reads per kb, normalized to 1 million sequenced miRNAs; PPM) mapping antisense to the *Tirant* consensus sequence in total RNA from ovaries and 0–30 min old embryos. Also shown are ping-pong and phasing Z-scores (equivalent *P* values: ns. equals not significant; * equals <0.05; ** equals <0.01; *** equals <0.001) for ovarian piRNAs. All ovarian small RNA data based on biological duplicates. (**B**) Density plots of sense (positive) and antisense (negative) *Burdock*-mapping piRNAs (PPM) along the *Burdock* consensus sequence in *B6* ovaries (left) and early embryos (right). Inset: 5′ overlap histogram of ovarian piRNAs (10-nucleotide ping-pong overlap in red). (**C**) As in (**B**), for strain *B4*. (**D**) Genomic copy numbers of *gypsy5* (left) estimated from genomic Illumina data and heatmaps (right) of antisense piRNA levels equal or longer than 23 nucleotides (thousand reads per kb, normalized to 1 million sequenced miRNAs; PPM) mapping antisense to the *Tirant* consensus sequence in total RNA from ovaries and 0–30 min old embryos. Also shown are ping-pong and phasing Z-scores (equivalent *P* values: ns. equals not significant; * equals <0.05; ** equals <0.01; ***equals <0.001) for ovarian piRNAs. All ovarian small RNA data based on biological duplicates. (**E**) Density plots of sense (positive) and antisense (negative) *gypsy5*-mapping piRNAs (PPM) along the *gypsy5* consensus sequence in *B6* ovaries (left) and early embryos (right). Inset: 5′ overlap histogram of ovarian piRNAs (10-nucleotide ping-pong overlap in red). (**F**) As in (**E**), for strain *B4*.

© The Author(s)

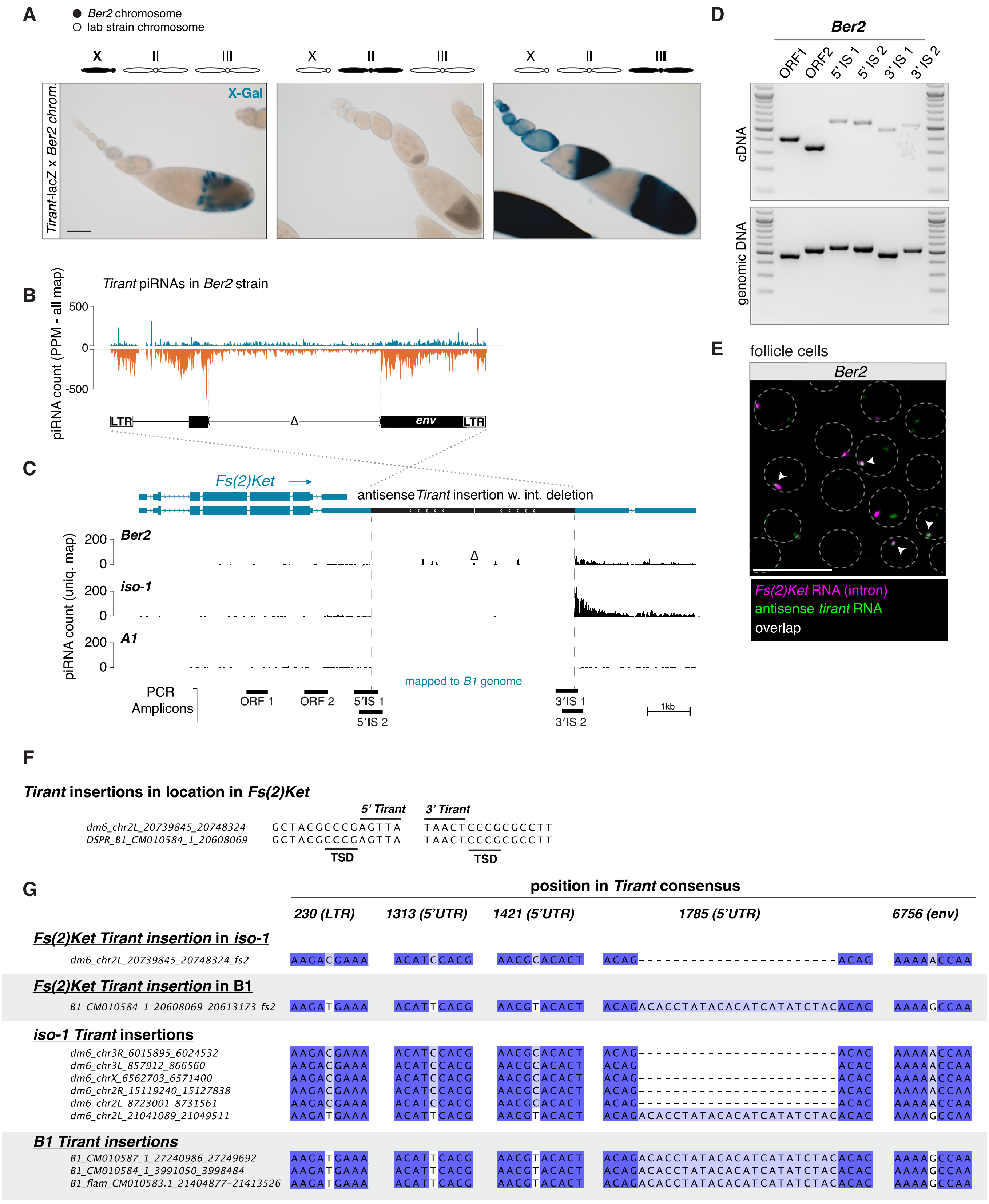
A
Ber2 chromosome
lab strain chromosome
X II III
Tirant-lacZ x Ber2 chrom.
X-Gal
B
Tirant piRNAs in Ber2 strain
piRNA count (PPM - all map)
500
0
-500
LTR
env
LTR
C
Fs(2)Ket
antisense Tirant insertion w. int. deletion
piRNA count (uniq. map)
Ber2
iso-1
A1
200
0
mapped to B1 genome
PCR Amplicons
ORF 1
ORF 2
5′IS 1
5′IS 2
3′IS 1
3′IS 2
1kb
D
Ber2
ORF1 ORF2 5′IS 1 5′IS 2 3′IS 1 3′IS 2
cDNA
genomic DNA
E
follicle cells
Ber2
Fs(2)Ket RNA (intron)
antisense tirant RNA
overlap
F
Tirant insertions in location in Fs(2)Ket
5′ Tirant
3′ Tirant
dm6_chr2L_20739845_20748324 GCTACGCCCGAGTTA TAACTCCCGCGCCTT
DSPR_B1_CM010584_1_20608069 GCTACGCCCGAGTTA TAACTCCCGCGCCTT
TSD
TSD
G
position in Tirant consensus
230 (LTR)
1313 (5′UTR)
1421 (5′UTR)
1785 (5′UTR)
6756 (env)
Fs(2)Ket Tirant insertion in iso-1
dm6_chr2L_20739845_20748324_fs2 AAGACGAAA ACATCCACG AACGCACACT ACAG----------------------ACAC AAAAACCAA
Fs(2)Ket Tirant insertion in B1
B1 CM010584 1 20608069 20613173 fs2 AAGATGAAA ACATTCACG AACGTACACT ACAGACACCTATACACATCATATCTACACAC AAAAGCCAA
iso-1 Tirant insertions
dm6_chr3R_6015895_6024532 AAGACGAAA ACATCCACG AACGCACACT ACAG----------------------ACAC AAAAACCAA
dm6_chr3L_857912_866560 AAGACGAAA ACATCCACG AACGCACACT ACAG----------------------ACAC AAAAACCAA
dm6_chrX_6562703_6571400 AAGACGAAA ACATCCACG AACGCACACT ACAG----------------------ACAC AAAAACCAA
dm6_chr2R_15119240_15127838 AAGACGAAA ACATCCACG AACGCACACT ACAG----------------------ACAC AAAAACCAA
dm6_chr2L_8723001_8731561 AAGACGAAA ACATCCACG AACGCACACT ACAG----------------------ACAC AAAAACCAA
dm6_chr2L_21041089_21049511 AAGATGAAA ACATTCACG AACGTACACT ACAGACACCTATACACATCATATCTACACAC AAAAGCCAA
B1 Tirant insertions
B1_CM010587_1_27240986_27249692 AAGATGAAA ACATTCACG AACGTACACT ACAGACACCTATACACATCATATCTACACAC AAAAGCCAA
B1_CM010584_1_3991050_3998484 AAGATGAAA ACATTCACG AACGTACACT ACAGACACCTATACACATCATATCTACACAC AAAAGCCAA
B1_flam_CM010583.1_21404877-21413526 AAGATGAAA ACATTCACG AACGTACACT ACAGACACCTATACACATCATATCTACACAC AAAAGCCAA

◀ **Figure EV2. A *Tirant* insertion in the *Fs(2)Ket* 3′ UTR is sufficient to produce piRNAs in the *B1/Ber2* strain.**

(**A**) X-gal stainings of ovarioles from the progeny of crosses between transgenic *Tirant-lacZ* reporter females and males harboring either the X (left), 2nd (middle), or 3rd chromosome (right) from the *Ber2* strain, as indicated. Scale bar: 100 µm. (**B**) Density plot of piRNAs (PPM) mapping to the *Tirant* consensus sequence isolated from *Ber2* ovaries. Dashed lines mark the region with distinctly lower piRNA coverage, corresponding to the deletion in the *Tirant* insertion within *Fs(2)Ket*. (**C**) UCSC browser screenshot of the *Fs(2)Ket* locus in the *B1* genome. The three tracks show genome-unique piRNAs (in PPM) that were sequenced from ovaries of *Ber2* (top), *iso-1* (middle), and *A1* (bottom), each mapped to the *B1* long-read genome assembly. piRNAs mapping uniquely to the internal deletion of *Tirant* are labeled by Δ. Relative genomic positions of analytic PCR amplicons shown in panel D are indicated at the bottom. Scale bar: 1 kb. (**D**) RT-PCR on RNA and PCR on genomic DNA from the *Ber2* strain to detect chimeric transcripts between *Fs(2)Ket* and *Tirant*. Positions of PCR amplicons are shown in panel C. Marker: 100 bp DNA ladder. (**E**) RNA-FISH detecting *Fs(2)Ket* (intronic probes; magenta) and antisense *Tirant* (green) transcripts in stage 8 egg chambers of the *Ber2* strain. Circumferences of follicle cell nuclei are marked by dashed lines. Arrowheads indicate foci where both signals co-localize. Scale bar: 20 µm. (**F**) Insertion site sequences of *Tirant* copies in *Fs(2)Ket* in the *iso-1* and *B1* strains. The target site duplications (TSD) flanking the *Tirant* LTRs from both 5′ and 3′ ends are indicated. (**G**) Sequence alignment showing informative parts of the *Tirant* insertions in the *Fs(2)Ket* 3′ UTR present in *iso-1* or *B1* together with a subset of other *Tirant* insertions in these strains. Shown are all SNPs and an insertion in the 5′ UTR that differ between the insertions in *iso-1* and *B1*. Source data are available online for this figure.

© The Author(s)

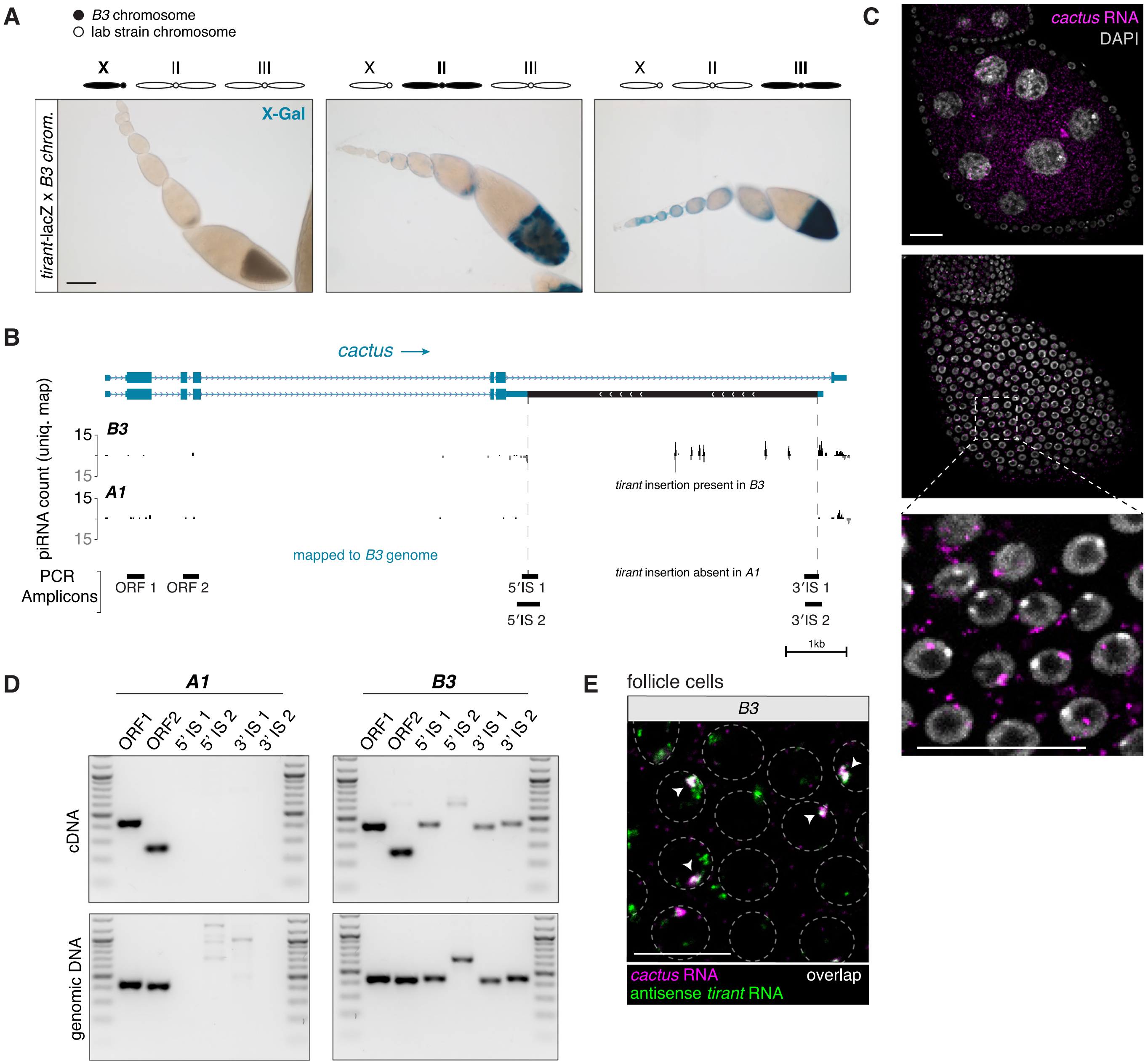

Figure EV3. A *Tirant* insertion in the *cactus* 3′ UTR is sufficient to produce piRNAs in the *B3* strain.

(**A**) X-gal stainings of ovarioles from the progeny of crosses between transgenic *Tirant-lacZ* reporter females and males harboring either the X (left), 2nd (middle), or 3rd chromosome (right) from the *B3* strain. Scale bar: 100 µm. (**B**) Genome-unique piRNAs mapping to the *cact* locus in the *B3* genome showing the two annotated *cact* isoforms. piRNAs from *A1* are shown as a negative control. Genomic positions of analytical PCR amplicons shown in panel D are indicated at the bottom. Scale bar: 1 kb. (**C**) RNA-FISH detecting *cact* transcripts (magenta) in the germline (top) or somatic cells (middle) of a Stage 6 egg chamber from the *A1* stain, with zoom-in from the boxed part shown below. DNA staining (DAPI) shown in gray. Scale bar: 20 µm. (**D**) RT-PCR on cDNA and PCR on genomic DNA from the *A1* and *B3* strains to detect chimeric transcripts between *cact* and *Tirant*. Positions of PCR amplicons are shown in (**C**). Marker: 100 bp DNA ladder. (**E**) RNA-FISH detecting *cact* (magenta) and antisense *Tirant* (green) transcripts in follicle cells of ovaries from the *B3* strain. Circumferences of follicle cell nuclei are marked by dashed lines. Arrowheads indicate foci where both signals co-localize. Scale bar: 20 µm. Source data are available online for this figure.

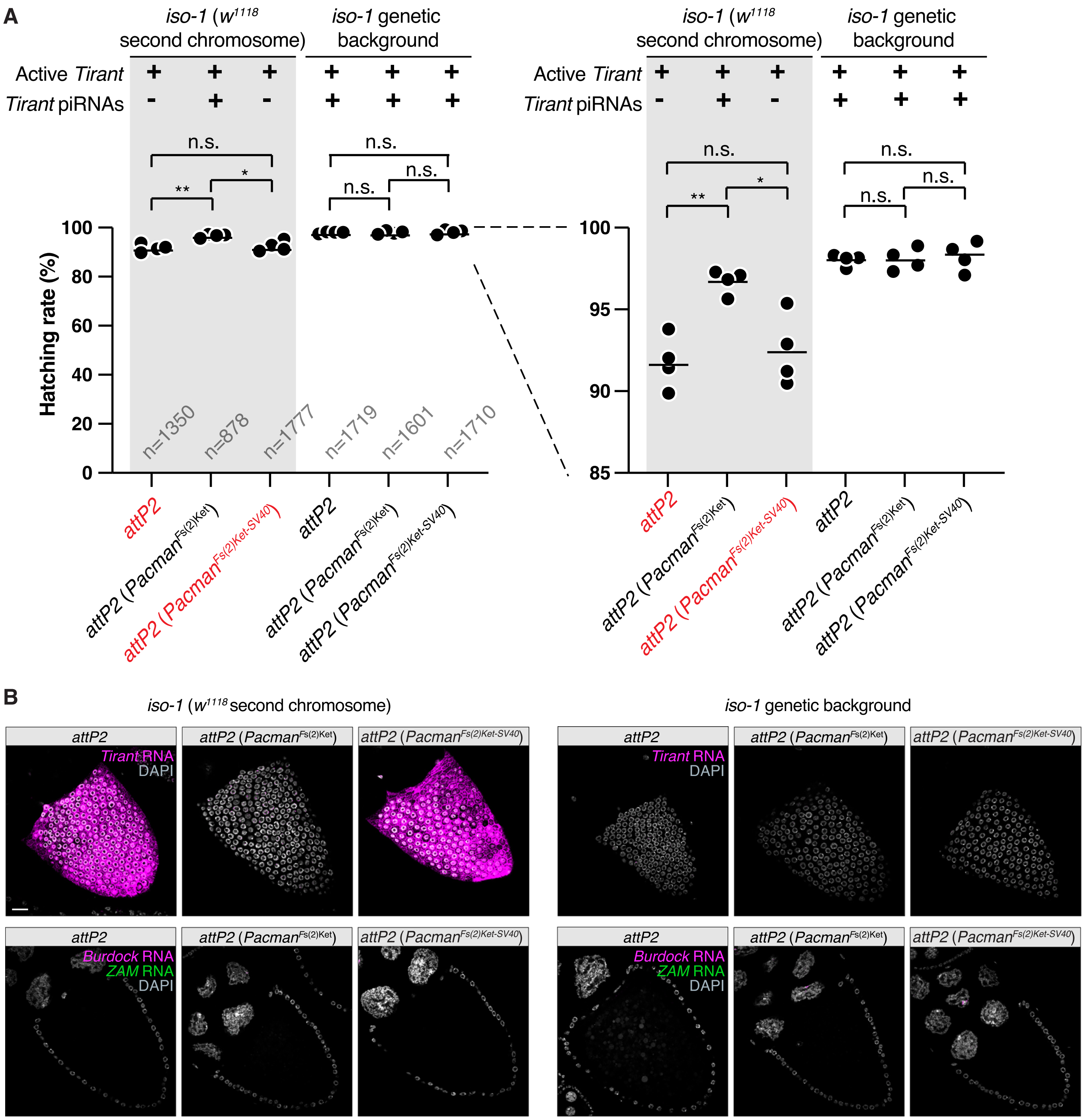

Figure EV4. The *Fs(2)Ket*^{Tirant} transgenic allele confers a significant fertility advantage to females expressing active *Tirant*.

(**A**) Hatching rate of eggs laid by females from crosses between females with indicated genotype (top) and males with indicated genotype (bottom). Each dot represents the mean of three egg collections with the exception of the *iso-1;w1118;iso-1* females containing the *Fs(2)Ket* transgene in *attP2* which was measured in duplicate. Genotypes shown in red indicate strains expressing active *Tirant* (see below). Total egg numbers analyzed are indicated for each genotype. Statistical significance was assessed using an paired two-sided *t* test (ns, not significant; $^{*}P < 0.05$; $^{**}P < 0.01$; $^{***}P < 0.001$. In *iso-1* (*w*^{1118} second chromosome) genetic background, *attP2* vs *attP2(Pacman*$^{Fs(2)Ket}$) $P = 0.0073$, *attP2* vs *attP2(Pacman*$^{Fs(2)Ket\text{-}SV40}$) $P = 0.4144$, *attP2(Pacman*$^{Fs(2)Ket}$) vs *attP2(Pacman*$^{Fs(2)Ket\text{-}SV40}$) $P = 0.0395$. In *iso-1* genetic background, *attP2* vs *attP2(Pacman*$^{Fs(2)Ket}$) $P = 0.9046$, *attP2* vs *attP2(Pacman*$^{Fs(2)Ket\text{-}SV40}$) $P = 0.7225$, *attP2(Pacman*$^{Fs(2)Ket}$) vs *attP2(Pacman*$^{Fs(2)Ket\text{-}SV40}$) $P = 0.7008$, $n = 4$, corresponding to 4 egg hatching counts for each condition). Bars indicate the mean. (**B**) RNA-FISH detecting *Tirant* sense transcripts (magenta – top) and *Burdock* and *ZAM* sense transcripts (magenta and green, respectively) in late-stage egg chambers from the strains used in the fertility assay above. DNA staining (DAPI) is shown in gray. Scale bar: 20 µm. Source data are available online for this figure.

 © The Author(s)

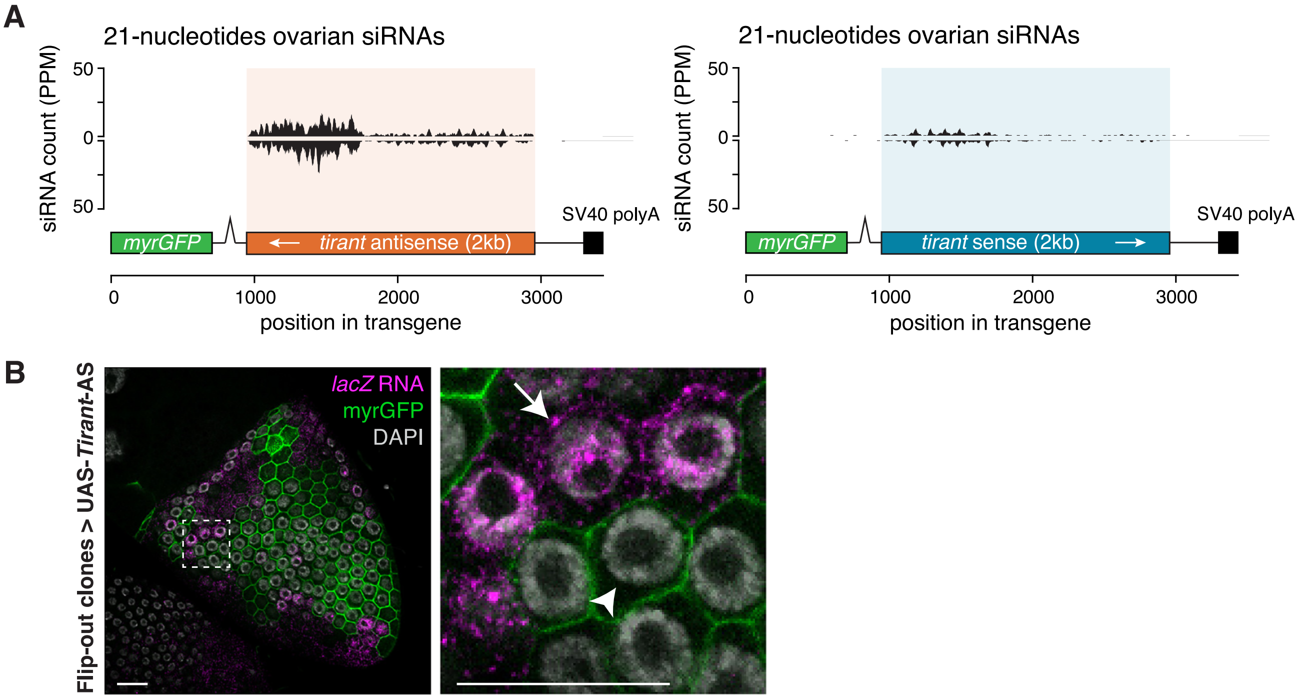

Figure EV5. An antisense fragment in the 3′ UTR of a UAS transgene results in siRNA production.

(**A**) Density plot of siRNAs (reads per 1 million sequenced miRNAs; PPM) sequenced from Argonaute-bound small RNAs extracted from ovaries expressing the indicated UAS-myrGFP-*Tirant* constructs by *tj*-Gal4. (**B**) RNA-FISH detecting *lacZ* transcript of the *Tirant* reporter (magenta) in the stage 10B egg chamber used in Fig. 8C (left panel) with a heatshock-induced flip-out clone expressing the UAS-myrGFP-*Tirant* antisense construct. Clones are marked by the presence of GFP (green). Right panel is a zoom-in from the boxed part in the left panel. Arrowhead indicates a cell inside the clone in which no transcripts are detected. The arrow indicates a cell outside the clone in which both cytoplasmic and nuclear transcripts are detected. DNA staining (DAPI) is shown in gray. Scale bar: 20 µm.

© The Author(s)
